# Modification of Pathological Nodal Classification for pT1b Esophageal Squamous Cell Carcinoma With Lymphovascular Invasion: Over 10‐Year Experience

**DOI:** 10.1002/cnr2.70342

**Published:** 2025-10-01

**Authors:** Jin‐bo Li, Li‐Hong Zhang, Chang‐Sen Leng, Jun‐Ying Chen, Jian‐Hua Fu

**Affiliations:** ^1^ Department of Thoracic Oncology Sun Yat‐sen University Cancer Center Guangzhou China; ^2^ State Key Laboratory of Oncology in South China, Collaborative Innovation Center for Cancer Medicine, Sun Yat‐sen University Cancer Center Guangzhou China; ^3^ Department of Pathology Sun Yat‐sen University Cancer Center Guangzhou China; ^4^ Guangdong Esophageal Cancer Institute Guangzhou China

**Keywords:** esophageal squamous cell carcinoma, lymphovascular invasion, pathological T1b, prognosis

## Abstract

**Background:**

Lymphovascular invasion (LVI) adversely affects the survival of pT1b esophageal squamous cell carcinoma (ESCC). It is hypothesized that a modified stage classification of pT1b ESCC based on LVI may facilitate multidisciplinary therapy in LVI‐positive patients.

**Aims:**

The study aims to investigate the impact of LVI on pathological nodal classification for pT1b ESCC.

**Methods and Results:**

Surgically resected pT1b ESCC patients in Sun Yat‐sen University Cancer Center between 2008 and 2018 were retrospectively reviewed. Tumor sections were re‐assessed for LVI by gastrointestinal pathologists. The associations between patient survival and LVI were evaluated by the Log‐rank method. A multivariate Cox regression model was applied to identify the impact of LVI on survival. Prognostic performance was assessed by Harrell's *C*‐index. A total of 424 cases with the pT1b stage were included. The risk of LVI was significantly higher in patients with nodal positive status (*p* < 0.001) and larger tumor size (*p* = 0.033). The 5‐year OS for LVI+ patients were 50.3% versus 78.0% for LVI− (*p* < 0.001). Multivariable analyses suggested that LVI (*p* = 0.021) and pN (*p* = 0.016) stages were two independent adverse prognostic factors in pT1b patients. When classifying LVI+ as an independent subgroup into the pN category, the modified pN staging system demonstrated a superior prognostic performance (*p* < 0.001).

**Conclusion:**

Tumors with LVI should be defined as a separate subclassification to accurately classify the prognostic category in pT1b patients. Further studies are required to investigate multidisciplinary therapies for LVI+ pT1b patients.

## 1 Introduction

Esophageal cancer is the sixth leading cause of cancer‐related death, and about 90% are esophageal squamous cell carcinoma (ESCC) [1]. The NCCN (National Comprehensive Cancer Network) guideline for esophageal and esophagogastric junction cancers provided the TNM stage system for optimizing the prognostic stratification and treatment selection [2]. In general, early‐stage ESCC has a superior prognosis, whereas the survival of patients would be worse with the increased stages. Nevertheless, some patients in the early stage still had a higher risk of developing rapid tumor relapse, which significantly shortened the length of survival [3]. Studies suggested that the depth of tumor infiltration, tumor size, and differentiation were potential risk factors of lymph node metastasis (LNM) in pathological T1 (pT1) patients and were associated with dismal survival [4, 5]. The issue that heterogeneities of prognosis exist in early‐stage ESCC requires further detailed stratification based on the NCCN TNM stage system [6].

Accurate tumor staging was pivotal for optimizing treatment selection. Surgery remained the mainstream for ESCC treatment; however, multidisciplinary therapies, including adjuvant chemotherapy, radiotherapy, and chemoradiotherapy, were also crucial for improving the prognosis of resectable ESCC with positive LNM. Generally, surgery alone would be adequate for early‐stage ESCC [7]. However, studies found that a subgroup of pT1 patients suffered from lower survival with surgery alone [8, 9]. The heterogeneities of prognosis may hint at considering adjuvant treatments rather than surgery alone in pT1 patients.

According to NCCN guidelines for esophageal and esophageal junction cancer, endoscopic submucosal resection is not recommended for T1b patients with lymphovascular invasion (LVI) [10]. LVI served as a factor to distinguish the subclassification of pT1b esophageal adenocarcinoma [11]. Above all, we hypothesized that a modified staging classification of pT1b ESCC based on LVI states may guide advanced multidisciplinary therapy. Indeed, the phenomenon of LVI can be effectively evaluated by normal light microscopic examination. Hence, we investigate the impact of LVI on survival and staging accuracy based on reviewing the LVI states and clinicopathological features in a large cohort of pT1b ESCC patients.

## 2 Methods

### 2.1 Patient Selection

Between January 2008 and January 2018, 1903 ESCC patients underwent esophagectomy, of whom 424 (35.3%) were pT1b and received two‐field lymph node dissection in the thoracic surgery department of Sun Yat‐sen University Cancer Center. The Mckeown or Ivor‐Lewis's procedure was applied for tumors located in the middle third or lower third of the thoracic esophagus. The work has been reported in line with the STROBE criteria [12].

Routine preoperative evaluations include history and physical examinations, blood tests, pulmonary and cardiac evaluations, neck, chest, and upper abdominal contrast computed tomography (CT), and upper gastrointestinal endoscopy with biopsy. The characteristics including age, gender, BMI (Body Mass Index), comorbidities, personal second malignancy, postoperative pathological stages, margin status, tumor size, LVI states, postoperative complications, neo‐adjuvant or adjuvant treatments, and follow‐ups were collected. All tumors and positive lymph nodes were re‐staged according to the seventh edition of the American Joint Committee on Cancer (AJCC) guideline. The retrospective study was approved by the IRB (Institutional Review Board) of Sun Yat‐sen University Cancer Center.

### 2.2 Pathological Assessment of LVI

The postoperative samples were processed by gastrointestinal pathologists according to the standardized procedures. LVI was assessed using hematoxylin and eosin (H&E) staining. Elastin Van Gieson (EVG) and D2‐40 immunostaining were not routinely performed due to institutional protocols prioritizing H&E for LVI identification, as validated in prior studies [13]. Tumor sections were evaluated across 5–10 slides per case, covering the entire tumor area. LVI was identified if tumor cells were observed in the vessel lumen (both lymphatic and blood vessel) with attachment to the vessel wall. Pathological T1b stage and lymph node classifications were identified according to the TNM staging system of NCCN guidelines.

### 2.3 Follow‐Up

Patients were followed every 3 months in the first 2 years, every 6 months for postoperative 3 to 5 years, and then annually. The routine follow‐up evaluations include blood tests, contrast CT, and upper gastrointestinal endoscopy. Blood tests included complete blood count, liver function tests, and tumor markers (CEA, SCC‐Ag) to monitor recurrence. In addition, the bone scan, brain MRI, or PET/CT would be applied if recurrence or metastasis was suspected.

### 2.4 Statistical Analysis

Statistical analysis was performed using R software version 3.6.3. The distributions of clinical categorical variables were compared by the Chi‐square test. The appropriate cut‐off point of tumor size was determined by the receiver operating characteristics curve (ROC). The tumor size cut‐off (2.1 cm) was selected via ROC analysis to maximize sensitivity and specificity for LVI prediction (AUC = 0.62, *p* = 0.02). Kaplan–Meier method with log‐rank test was used to calculate the survival curves. Collinearity diagnosis was applied if interdependence between variables was identified by Chi‐square test. The multivariate survival analysis was calculated by the Cox regression method. Harrell concordance index (*C*‐index), Akaike information criterion (AIC), and likelihood ratio test were used to evaluate the performance of prognostic models. All tests were two sided, and a *p*‐value less than 0.05 was considered statistically significant.

## 3 Results

### 3.1 Baseline Patient and LVI Characteristics

A total of 424 patients diagnosed as pT1b stage ESCC who underwent R0 resections were included. More LVI+ patients received adjuvant therapy (43.2% vs. 15.5%, *p* < 0.001) due to higher LNM rates (65.9% vs. 21.8%, *p* < 0.001), per institutional protocols for high‐risk cases. Tumor size with the cut‐off value of 2.1 cm had the greatest area under the ROC curve (*p* = 0.02, Figure S1) and was significantly related to LVI (*p* = 0.033, Table 1). LVI was not correlated with patient age and gender. Moreover, the distribution of smoking, alcohol consumption, number of inspected lymph nodes, or family tumor history had no difference in terms of LVI state.

**TABLE 1:** The distribution of clinical characteristics in pT1b ESCC patients with or without LVI.

Characteristics	Groups		*p*
	LVI (*N* = 44)	Non‐LVI (*N* = 380)	
Age (mean ± SD)	60.5 ± 6.5	60.0 ± 8.0	0.631
Gender (M/F, %)	32 (72.7)/12 (27.3)	284 (74.7)/96 (25.3)	0.772
Smoking (yes/no, %)	25 (56.8)/19 (43.2)	172 (45.3)/208 (54.7)	0.146
Alcohol (yes/no, %)	17 (38.6)/27 (61.4)	114 (30.0)/266 (70.0)	0.241
Family tumor history (yes/no, %)	7 (15.9)/37 (84.1)	58 (15.3)/322 (84.7)	0.910
Tumor size (≤ 2.1 cm/> 2.1 cm)	24 (54.5)/20 (45.5)	267 (70.3)/113 (29.7)	0.033
Number of inspected lymph Nodes (mean ± SD)	26.7 ± 14.7	24.7 ± 15.0	0.405
pN stages (N0/N+, %)	15 (34.1)/29 (65.9)	297 (78.2)/83 (21.8)	< 0.001
G stages (G1, *x*/G2/G3, %)	1 (2.3)/24 (54.5)/19 (43.2)	21 (5.5)/228 (60.0)/131 (34.5)	0.402
Neoadjuvant therapy (yes/no, %)	2 (4.5)/42 (95.5)	22 (5.8)/358 (94.2)	0.735
Adjuvant therapy (yes/no, %)	19 (43.2)/25 (56.8)	59 (15.5)/321 (84.5)	< 0.001

Abbreviations: LVI, lymphovascular invasion; N+, nodal positive; non‐LVI, non‐lymphovascular invasion; SD, standard deviation.

### 3.2 Survival Impact of LVI in pT1b ESCC Patients

**FIGURE 1:**
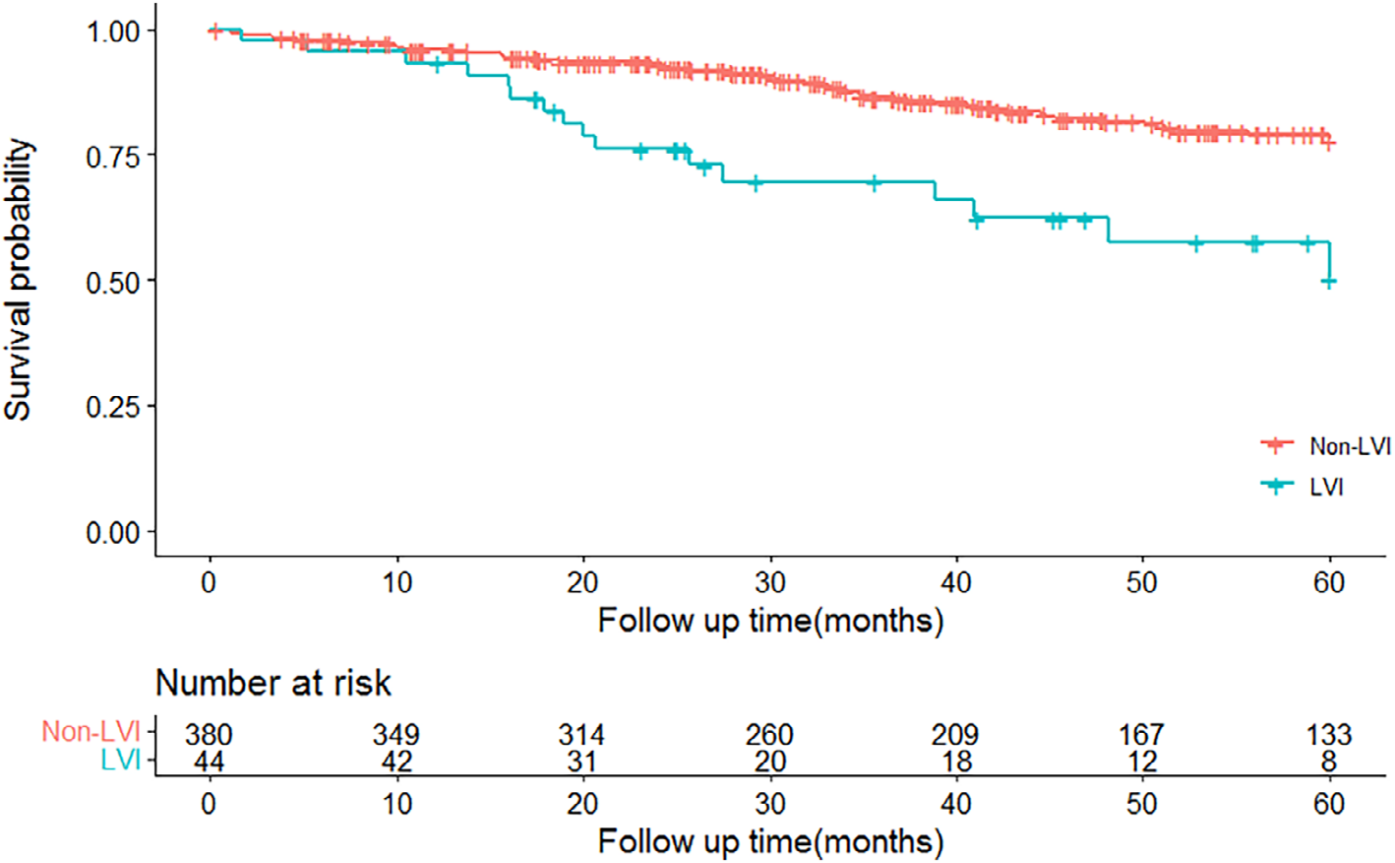
Overall survival curves of pT1b ESCC patients with or without LVI.

The median follow‐up time was 45.5 months, ranging from 1.1 to 143.8 months. Distant relapses were developed in 10 (22.7%) and 37 (9.7%) cases from LVI and non‐LVI groups, respectively (*p* = 0.019). To further analyze the survival impact of LVI, the 5‐year estimated overall survival (OS) rates were calculated in cases with or without LVI. The result of the log‐rank test showed a great survival difference, and the estimated 5‐year survival rate was 50.3% (95% CI, 33.7%–75.2%) and 78.0% (95% CI, 73.0%–83.2%), for T1b patients with or without LVI, respectively (*p* < 0.001, Figure 1).

### 3.3 Multivariable Cox Regression Analysis

Due to the interdependence between LVI, LNM (*p* < 0.001, Table 1), and tumor size (*p* = 0.033, Table 1), collinearity diagnosis was performed. As a result, the tolerance and VIF did not show any significant collinearity between the variables (Table S1).

In multivariable Cox regression analysis, factors such as LVI status, tumor size, tumor grade, smoking history, alcohol drinking history, family malignant tumor history, neo‐adjuvant therapy, and adjuvant therapy were included. Finally, LVI (HR 2.03, 95% CI 1.12–3.69, *p* = 0.021) and LNM (HR 1.82, 95% CI 1.12–2.96, *p* = 0.016) states showed independent adverse impacts on OS.

### 3.4 Superior Prognostic Model

**FIGURE 2:**
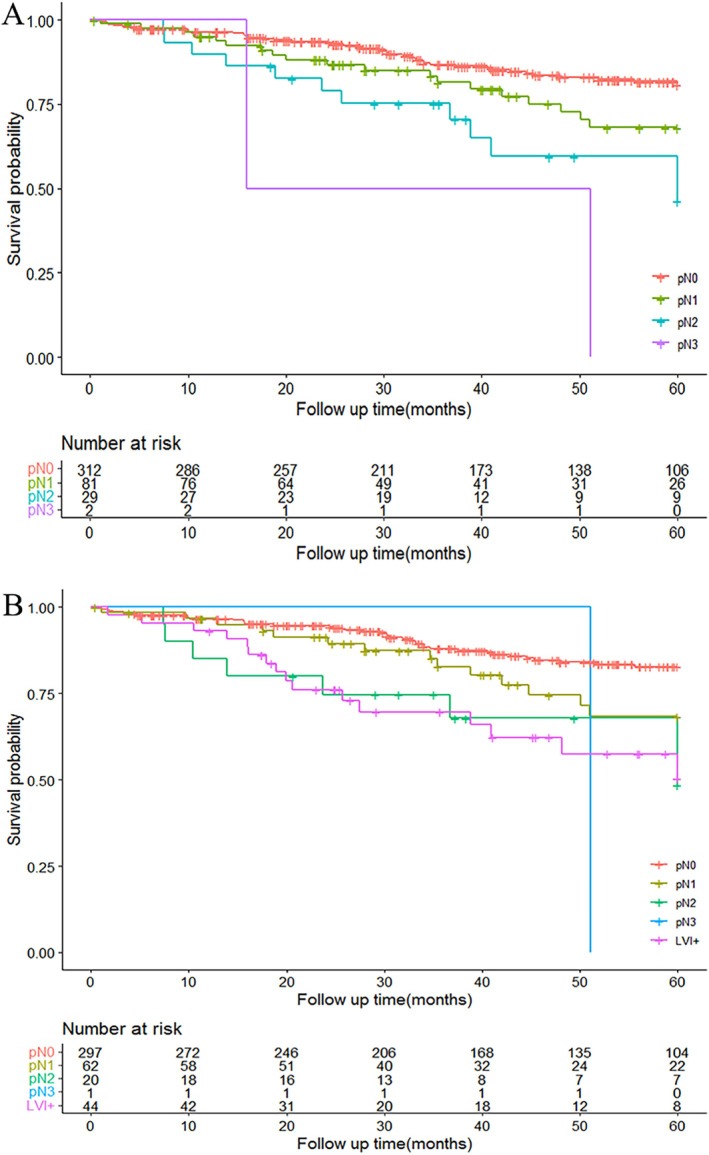
Survival curve of pT1b ESCC patients separated by pN and modified pN system. Survival curves of pT1b patients separated by TNM stages (A); survival curves of pT1b patients separated by modified pN stages with LVI+ as an independent prognostic subgroup (B).

The number of cases in the pN0, pN1, pN2, and pN3 categories was 312 (73.6%), 81 (19.1%), 29 (6.8%), and 2 (0.5%), respectively. The survival curves of pT1b cases according to staging classifications are shown in Figure 2A. When comparing different pN stages, the LVI+ subgroup had a significantly worse prognosis than the pN0 category (*p* = 0.001), while close to the LNM+ categories (Figure 2B).

The result indicated that LVI+ might be considered as a separate prognostic feature. When classifying LVI+ cases as an independent prognostic subgroup, the modified N staging showed superior prognostic performance with a higher *C*‐index (0.618 vs. 0.588, *p* < 0.001).

## 4 Discussion

Our study demonstrates that LVI is an independent adverse prognostic factor for OS in pT1b ESCC, with LVI+ patients exhibiting significantly worse 5‐year OS (50.3% vs. 78.0%, *p* < 0.001). This study does not propose altering the TNM system but highlights LVI as a critical adjuvant prognostic variable to refine risk stratification. Incorporating LVI into a modified N staging system improved prognostic accuracy (*C*‐index: 0.618 vs. 0.588, *p* < 0.001), suggesting its clinical utility for risk stratification.

The mechanism for histopathological correlation between LVI and LNM remained unclear. Nonetheless, studies suggested that LVI and LNM were not mutually independent. As our results, higher pN categories were correlated with a higher incidence of LVI. Correspondently, a previous study reported that LVI might be the pathological process prior to LNM in superficial ESCC [14]. Therefore, we hypothesized that LVI may act as a significant factor in the modified staging classification of pT1b ESCC. Similarly, research showed that LVI served as a factor in distinguishing the subclassification of pT1b esophageal adenocarcinoma [11]. Our collinearity analysis confirmed statistical independence between LVI and LNM (VIF < 2) in this cohort, suggesting distinct prognostic contributions. Clinically, these results advocate for routine LVI assessment in pT1b ESCC to guide adjuvant therapy, particularly in LVI+ patients who may benefit from intensified surveillance or multimodal interventions. Future studies should validate this model in larger cohorts and explore molecular mechanisms underlying LVI‐driven tumor aggressiveness.

In addition, LVI has been reported as a prognostic factor in ESCC [15]. Moreover, LVI is independently associated with shorter OS in neo‐adjuvant chemoradiotherapy‐treated ESCC, and the prognostic effects of LVI are similar to LNM [16]. The adverse survival impact of LVI has been identified in other malignant diseases, such as upper urinary tract urothelial carcinoma [17], cervical cancer [18], prostate cancer [19], and Merkel cell carcinoma [20]. Due to the potential interdependence between LNM and LVI, further collinearity diagnosis was conducted, which ruled out mutual dependence between the two variables. Then the multivariate analysis showed that LVI and pN were two independent adverse factors for pT1b patients.

The tumor size records were retrospectively traced and analyzed in our cohort. But there are two major limitations in further clinical applications: some mucosal tumor lesions were not visible by eye, but could only be identified by microscope, and the tumor size was hard to measure accurately after neoadjuvant therapy. In addition, the max length of the lesion should be chosen as a measurement of tumor size to increase the accessibility in the pathological examination. The discrepancy in tumor size measurements might be the reason for different results on prognostic correlations.

Although the pT1b ESCC was much less than the advanced stages at diagnosis, we included a relatively large cohort of 424 patients. It is notable that survival heterogeneity was observed in pT1b esophageal cancer [3, 21]. However, the factors contributing to the worse prognosis in pT1b patients are still under investigation. Though the detailed histopathological mechanism for the worse prognostic effect of LVI is largely unknown, the strong correlation between LVI and LNM has been identified in different cancers [22, 23]. Therefore, it is possible that LVI may indicate an increased risk of LNM, or may like a “limited metastatic status.” Secondly, LVI may suggest a much more malignant tumor behavior. Schiefer et al. found that LVI can occur in the esophageal cancer primary site (52.1%), as well as metastatic lymph nodes (52.5%) and distant metastases (22.2%), and LVI states strongly related to occurrence of LVI in metastatic lymph node; moreover, the presence of LVI in the primary site and especially in lymph node was correlated with poor survival [24].

Adding LVI patients into a separate prognostic subgroup improved the accuracy of the survival prediction model. Harrell et al. introduced *C*‐index as a discriminative parameter for the evaluation of survival models [25]. By calculating *C*‐index, the relative risk order of different covariates between groups of patients could be compared. Therefore, the *C*‐index is widely used to evaluate the performance of survival models. AIC was also frequently used in selecting the most appropriate model [26]. A better *C*‐index was observed in the LVI‐modified pN staging system, demonstrating its superiority as a prognostic model compared to the TNM staging system. As much as we know, the study is the first one evaluating the performance of LVI in the prognostic model for pT1b ESCC. However, the number of cases in the LVI+ subgroup was relatively small; the prognostic model should be further identified in large‐scale analysis.

The findings have to be analyzed in light of limitations. Our study was solely focused on LVI at the primary tumor site. Further investigations are needed to clarify the clinical significance of LVI in metastatic lymph nodes and distant metastases. Additionally, the retrospective nature of this study inherently imposes certain limitations. Future trials may consider focusing on multidisciplinary treatment strategies for pT1b patients, particularly those with SM1 infiltration who undergo endoscopic submucosal resection.

## 5 Conclusions

In conclusion, pT1b ESCC with LVI should be defined as a separate prognostic subclassification. Our findings advocate for routine LVI assessment in clinical practice to identify high‐risk patients who may benefit from adjuvant therapies, aligning with prior evidence on LVI's role in early‐stage cancers. Future prospective studies are warranted to validate this model in larger cohorts and elucidate the molecular mechanisms linking LVI to tumor aggressiveness, ultimately refining personalized treatment strategies for pT1b ESCC.

## Author Contributions

Jin‐bo Li contributed to data curation, investigation, methodology, and software. Li‐Hong Zhang contributed to software, methodology, writing original draft, and validation. Chang‐Sen Leng contributed to validation. Jun‐Ying Chen contributed to conceptualization, and writing original draft. Jian‐Hua Fu contributed to conceptualization, visualization, and writing review and editing.

## Ethics Statement

This study has been approved by the IRB of Sun Yat‐sen University Cancer Center (ID: B2023‐574‐01).

## Consent

According to the IRB committee, patient consent for publication has been waived.

## Conflicts of Interest

The authors declare no conflicts of interest.

## Supporting information


**Figure S1:** ROC curve showing the cut‐off value of tumor size.

**Table S1:** The tolerance and VIF on collinearity between the variables.

## Data Availability

The data and material would be available upon reasonable request.
