# Supplementary figures and images for: Modification of Pathological Nodal Classification for pT1b Esophageal Squamous Cell Carcinoma With Lymphovascular Invasion: Over 10‐Year Experience
Source: Cancer Rep (Hoboken). 2025 Oct 1;8(10):e70342. doi: 10.1002/cnr2.70342 (PMC12485821; doi:10.1002/cnr2.70342)

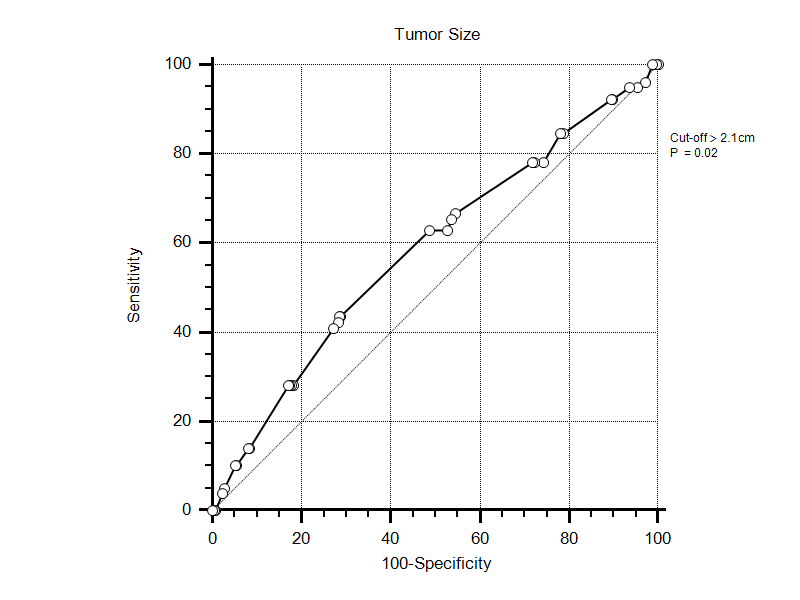

Supplement: Supplementary file 1 — Figure S1: ROC curve showing the cut‐off value of tumor size. [file CNR2-8-e70342-s002.tif]
