# Supplementary material for: Modification of Pathological Nodal Classification for pT1b Esophageal Squamous Cell Carcinoma With Lymphovascular Invasion: Over 10‐Year Experience
Source: Cancer Rep (Hoboken). 2025 Oct 1;8(10):e70342. doi: 10.1002/cnr2.70342 (PMC12485821; doi:10.1002/cnr2.70342)
Supplement: Supplementary file 2 — Table S1: The tolerance and VIF on collinearity between the variables. [file CNR2-8-e70342-s001.docx]

Table S1: Results of collinearity diagnosis

| Variables | Tolerance | VIF |
| --- | --- | --- |
| LVI | 0.902 | 1.109 |
| pN stages | 0.886 | 1.129 |
| Tumor size | 0.969 | 1.032 |
